# The Infectious Diseases Reports

**Published:** 1920-09-18

**Authors:** 


### THE INFECTIOUS DISEASES REPORTS.

Both the second report of the Ministry of Health on public health and medical subjects entitled "The Incidence of Notifiable Infectious Diseases in Each Sanitary District in England and Wales during the Year 1919," and the more recent M.A.B. and Health Ministry Annual Reports throw some interesting light upon the latest occurrences of these maladies, but as has invariably occurred since 1914, the everchanging conditions of the war period have so complicated the statistical part of such observations, and have brought in so many possible fallacies that we must not be too ready to accept the figures as truly representative of the facts.

Of late the subject of the epidemiology and etiology has been frequently to the fore, and it is interesting to compare these published records in the light of Dr. Hamer's contention that there is a direct connection between scarlet fever and fleas. It will be remembered that both 1913 and 1914 were notorious years for this disease, and that these epidemics were followed by a decline to the low point of about 1.5 per thousand of population during 1917 and 1918, while the death-rate fell to the lowest recorded in this country. Now, this was remarkable considering that at this period, apart from the ravages of influenza, there was much general lowering of the bodily resistance of the home population, and that other diseases, such as cerebro-spinal fever, encephalitis, and diphtheria, all appeared to take advantage of the situation. We must admit that with the population under more or less constant movement and renewed supervision, to say nothing of a general cleansing of both premises and people by the manifold agencies of the Army and munition-making requirements, there must inevitably have been a great reduction in the number of fleas abounding during 1916, 1917, and 1918, and it seems that Dr. Hamer's assertion that fewer lodging-house beds showed flea-markings during this period of the war fits in perfectly with the probabilities. Also, as conditions of crowding gradually revert to their former state, it is interesting to note that last year during the winter there was a definite and rapid increase in the cases of scarlet fever, although the severity of the outbreak was relatively mild. In any case it is with these "simple" and well-known diseases that the greatest need for investigational work lies, and we see that this survey of their occurrence, extending over periods including a sudden change of social conditions, a period of permanent organised and controlled public existence, followed by a gradual reversion more or less completely to the original state of typical urban life, should provide some remarkably valuable information. We look forward with interest to the next report and figures, which will probably include the whole of those times historically to be connected with the Great War.

One fact, surprising perhaps to those who have taken for granted that the recent boom in health administration had already brought about its promised results, lies in the report that several diseases show an increase over the figures of 1918. In many cases this is sufficiently marked that it probably cannot be accounted for by more thorough notification, and the increased number of people who have come again under the care of the civil authorities. Apart from scarlet fever, we note in this respect diphtheria, puerperal fever, erysipelas, cerebro-spinal fever, poliomyelitis, and ophthalmia neonatorum.

With reference to the recently instituted notifications, it is interesting to observe that no fewer than 45,260 cases of pneumonia were recorded in 1919. Also we read of 1,657 cases of dysentery, 99 cases of trench fever, and 14,483 of malaria, although, as we have previously explained, the majority of the last-mentioned have certainly occurred in soldiers returned from abroad.

